# Calibrating EASY-Care independence scale to improve accuracy

**DOI:** 10.1093/ageing/afw106

**Published:** 2016-11-02

**Authors:** A. T. Jotheeswaran, Amit Dias, Ian Philp, Vikram Patel, Martin Prince

**Affiliations:** 1 Indian Institute of Public Health, Hyderabad, Hyderabad, Telangana 500033, India; 2 Institute of Psychiatry, King's College London, London SE58AF, UK; 3 Department of Preventive and Social Medicine, Goa Medical College, Goa, India; 4 Hull and East Yorkshire Hospitals NHS Trust, Hull, UK; 5 London School of Hygiene and Tropical Medicine, Centre for Global Mental Health, London, UK; 6 Sangath, Goa, India; 7 Health Service and Population Research Department, Institute of Psychiatry, Psychology & Neuroscience, and Centre for Global Mental Health, King's College London, London, UK

**Keywords:** dependence, independence, ageing, India, dependence scale, older people

## Abstract

**Background:**

there is currently limited support for the reliability and validity of the EASY-Care independence scale, with little work carried out in low- or middle-income countries. Therefore, we assessed the internal construct validity and hierarchical and classical scaling properties among frail dependent older people in the community.

**Objective:**

we assessed the internal construct validity and hierarchical and classical scaling properties among frail dependent older people in the community.

**Methods:**

three primary care physicians administered EASY-Care comprehensive geriatric assessment for 150 frail and/or dependent older people in the primary care setting. A Mokken model was applied to investigate hierarchical scaling properties of EASY-Care independence scale, and internal consistency (Cronbach's alpha) of the scale was also examined.

**Results:**

we found that EASY-Care independence scale is highly internally consistent and is a strong hierarchical scale, hence providing strong evidence for unidimensionality. However, two items in the scale (unable to use telephone and manage finances) had much lower item Loevinger H coefficients than others. Exclusion of these two items improved the overall internal consistency of the scale.

**Conclusions:**

the strong performance of the EASY-Care independence scale among community-dwelling frail older people is encouraging. This study confirms that EASY-Care independence scale is highly internally consistent and a strong hierarchical scale.

Care dependence is an important clinical outcome for older people and healthcare providers [1]. A shift from independence to dependence is conventionally measured using activities of daily living (ADL) or instrumental activities of daily living (IADL) scales [2]. Hierarchical scaling properties, where constituent items have different inherent ‘item difficulties’, confer several desirable properties for measurement efficiency, including unidimensionality and simplified scaling metrics (the sum of the unweighted item scores approximating to the position on the underlying latent trait)—these properties have been demonstrated for several widely used scales in this domain of assessment [3]. The approach taken for the widely used EASY-Care scale was somewhat different, being based essentially on classical scaling principles, with items weighted to reflect the presumed significance of endorsement for overall severity.

The EASY-Care independence scale was originally developed from the Barthel Index and the Duke OARS IADL Scale [4]. It consists of 18 items ascertaining limitations in ADL and IADL. The weighted items include use of telephone, keeping up appearance, dressing, bathing, housework, preparing meals, feeding, taking medications, urinary incontinence, faecal incontinence, ability to use the toilet, transferring from bed to chair, mobility inside the home, managing stairs, mobility outside home, ability to shop, use of public services and managing finances [5]. The total score ranges from 0 to 100, with higher scores denoting greater degree of dependence and need for care.

There is currently limited support for the reliability and validity of the EASY-Care independence scale, with little work carried out in low- or middle-income countries. Test–retest reliability at item level was adequate among 50 patients attending a UK geriatric rehabilitation facility [5]. Face validity of individual items was established in the UK/USA cross-national consensus of professionals and older service users, although the importance attached to the items varied between these two groups [6]. The feasibility and utility of the scale was supported through evaluations of patients’ and clinicians’ experience in Colombia, Kerala, Lesotho, Tonga, Iran and UK [6]. The unidimensionality of the EASY-Care independence scale has not been empirically tested, neither is it clear whether it has hierarchical scaling properties [7]. Therefore, given the salience of the EASY-Care independence scale, we assessed the internal construct validity and hierarchical and classical scaling properties among frail dependent older people in the community.

## Methods

This study was conducted in a primary healthcare setting in Goa, India. Detailed information on participants’ recruitment is described elsewhere [8]. Ten community health workers identified 152 frail and/or dependent older people at the community level and assessed their needs for care using methods developed by the 10/66 Dementia Research Group [9]. Intervals of care were rated in seven bands from ‘cannot be left on their own’ to ‘more than 3 days’; intensity of care was rated as ‘no needs for care’, ‘needs care occasionally’ and ‘needs care much of the time’. Mobility restriction was rated in five bands from ‘fully mobile outside of the home’ to ‘bedbound’. Three primary healthcare physicians reassessed the older people with EASY-Care assessment. This study was conducted between 2013 and 2014. King's College Research Ethics Committee and Institutional Ethics Committee of Public Health Foundation of India approved the study.

## Statistical analysis

Internal consistency (Cronbach's alpha) for the EASY-Care independence scale was calculated using SPSS 21.0 [10]. A Mokken model was applied to investigate hierarchical scaling properties using STATA 11.0 after downloading the LoevH add-on programme from http://www.anaqol.org. Mokken scaling involves the application of a non-parametric item response model [11] to measure the hierarchical properties of items in a scale, assessing if the items can be ordered by degree of difficulty, such that any individual who endorses a particular item will also endorse all the items ranked lower in difficulty. Three basic assumptions are required for a monotone homogeneity model: (i) unidimensionality (one latent variable summarises the variation in the item scores in the questionnaire), (ii) local independence (after conditioning on the position on the latent trait, the item scores are statistically independent) and (iii) monotonicity (for all items, the probability of a positive response increases monotonically with increasing values of the latent trait). These assumptions being met, an individual's position on the latent trait can conveniently be estimated as the rank of the highest item in the hierarchy that they endorse, or their total number of positive responses [12].

In addition, double monotonicity models was applied for values of the latent trait, to assess the probability of a positive to decrease with the difficulty of the item. This means that the order of item difficulties remains invariant over all values of the latent trait and thus, the item response function curves do not intersect [13,14]. To assess single monotonicity, we estimated Loevinger coefficients for each item (Hi) and for the whole scale (H), where values between 0.3 and 0.4 suggest weak scalability, values between 0.4 and 0.5 moderate and values above 0.5 strong scalability. We also tested formally for violations of monotonicity (using the Stata loevh monotonicity command) and non-intersection (using the Stata loevh nipmatrix command) between pairs of items (minimum violation 0.03, alpha = 0.05), using overall criteria values as an indication of the likelihood of assumption violation; ≤40 ‘satisfactory’, 40–79 ‘questionable violation’, ≥80 ‘strongly suggesting an assumption violation’ [15]. Concurrent validity of the EASY-Care independence scale was assessed by estimating the variance in this outcome accounted for by the extent of the needs for care as assessed by a Community Health Worker (CHW) (intervals of care, intensity of care) and mobility restriction, using univariate general linear models.

## Results

Cronbach's alpha, reflecting internal consistency across the 18 EASY-Care independence scale items, was 0.89. Item and scale Loevinger H coefficients were estimated using a polytomous Mokken analysis. There was robust evidence that the EASY-Care independence scale and its constituent items conformed to a ‘strong’ Mokken scale (Table 1). The coefficient H values for individual items exceeded 0.47 (range 0.47–0.68) other than two items: unable to use telephone (0.27) and unable to manage finances (−0.13). The overall scale H coefficient was 0.50. There were no statistically significant violations of monotonicity assumptions. However, there were a number of statistically significant violations with respect to non-intersection (double monotonicity). Of these, only ‘unable to do housework’, ‘confined to bed’ and ‘unable to manage finances’ were associated with criteria values >80, strongly suggesting an assumption violation. Internal consistency of the items (Cronbach's alpha) was 0.88, providing further evidence of unidimensionality.

**Table 1.** Polytomous Mokken analysis with EASY-Care assessment independent scale

Items	Mean score	Loevinger H coefficient	Non-intersection (Pmatrices curve)
Unable to use telephone	2.3	0.27	78
Needs help with keeping up appearance	1.1	0.57	19
Unable to dress	1.2	0.67	47
Unable to bath	1.2	0.63	65
Unable to do housework	2.4	0.55	90
Unable to prepare meals	2.6	0.47	64
Unable to feed	1.3	0.47	61
Unable to take medicines	1.4	0.47	63
Urinary incontinence	1.3	0.55	67
Faecal incontinence	1.2	0.63	49
Unable to use toilet	1.3	0.68	64
Unable to move from bed to chair	1.3	0.53	69
Confined to bed	1.6	0.50	92
Unable to manage stairs	2.2	0.48	53
Unable to walk outside	2.0	0.52	37
Unable to shop	2.8	0.49	16
Unable to get public services	2.1	0.51	38
Unable to manage finances	1.6	−0.13	186
Overall scale Loevinger's H coefficienct		0.50	0.61
Cronbach's alpha (internal consistency of the items)	0.88		

## Concurrent validity

For the purposes of the analyses of concurrent validity, we used the weighted EASY-Care score, and then reassessed associations using the unweighted (raw) score. The correlation between weighted and unweighted scores was 0.990. Needs for care as identified by the CHW explained 35.9% of the variance in the independence scale score. Those identified with needs for care ‘much of the time’ had higher independence scale scores (mean 52.9, SD 21.3) than those with occasional needs for care (mean 30.1, SD 10.8) and those with no needs for care (mean 27.2, SD 8.6). Intervals of care as assessed by the CHW explained 21.4% of the variance in independence score, with scores increasing monotonically from the longest interval (more than 3 days, mean 24.2, SD 8.8) to the shortest (cannot be left alone, mean 48.1, SD 22.8). Mobility hierarchy explained 49.1% of the variance in independence score, with scores increasing monotonically from those with no mobility restriction (mean 23.2, SD 8.0) to those who were bedbound (mean 68.4, SD 22.3).

## Discussion

This study confirms that EASY-Care independence scale has robust measurement properties. A scale is unidimensional if all the items of the scale measure one common latent variable. Hierarchical scales have particularly desirable measurement properties in terms of precision and measurement efficiency. We found that EASY-Care independence scale is highly internally consistent and is a strong hierarchical scale, hence providing strong evidence for unidimensionality. Two items in the scale (unable to use telephone and manage finances) had much lower item Loevinger H coefficients than others. This is partly due to cultural appropriateness of the items in the scale. In India, it is common for older people to transfer financial management responsibilities to co-resident spouse or children after retirement [16]. In such a context, the question of inability to manage finances may be irrelevant, or at least less reliably discriminating than in other cultures where older people retain this role and responsibility other than in the context of incapacity. Likewise, telephone use is uncommon among older people particularly since telecommunications are generally conducted via personal mobile phones rather than fixed landlines [16]. The unidimensionality of the independence scale can be improved if these two items are dropped. Most earlier studies tested functional decline measure in selected community population and validity of ADL measure on frail older people is less investigated [17].

The strong performance of the EASY-Care independence scale among community-dwelling frail older people is encouraging, since this will be a useful confirmatory indicator of disability and needs for care.

**Key points**

- This study confirms that EASY-Care independence scale has robust measurement properties.
- High internal consistency and hierarchical nature provides evidence for unidimensionality.
- The strong performance of EASY-Care independence scale is encouraging, Since this will be a useful confirmatory indicator of disability and needs for care in frail older people living in the community.
